# Dynamic ^11^C-Choline PET / CT for the primary diagnosis of prostate cancer

**DOI:** 10.1590/S1677-5538.IBJU.2018.0035

**Published:** 2018

**Authors:** Shay Golan, Meital Nidam, Hanna Bernstine, Jack Baniel, David Groshar

**Affiliations:** 1 Institute of Urology, Rabin Medical Center - Beilinson Hospital, Petach Tikva, Sackler Faculty of Medicine, Israel; 2 Department of Nuclear Medicine, Rabin Medical Center - Beilinson Hospital, Petach Tikva, Sackler Faculty of Medicine, Israel

**Keywords:** Positron-Emission Tomography, Tomography, X-Ray Computed, Prostatic Neoplasms

## Abstract

**Objectives:**

To test the ability of dynamic ^11^C-PET / CT to discriminate cancerous tissue from background tissue in patients with localized prostate cancer.

**Materials and Methods:**

Twenty-four consecutive patients with prostate cancer were prospectively evaluated with dynamic ^11^C-choline PET / CT prior to radical prostatectomy. The PET / CT scan was divided into 18 sequences of 5 seconds each, followed by 9 sequences of 60 seconds each. Whole-mount sections of harvested prostates served as reference standards. Volumes of interest were positioned on the dynamic PET / CT images and the following quantitative variables were calculated: perfusion coefficient (K1), washout constant (K2), area under the curve (AUC) at 175 and 630 seconds, and average and maximum standardized uptake values (SUV_avg_, and SUV_max_). Wilcoxon signed-ranks test was used to compare benign and cancerous areas of the prostate.

**Results:**

Areas of cancerous tissue were characterized by higher SUV_avg_ and SUV_max_ than areas of benign tissue (3.67 ± 2.7 vs. 2.08 ± 1.3 and 5.91 ± 4.4 vs. 3.71 ± 3.7, respectively, P < 0.001), in addition to a higher K1 (0.95 ± 0.58 vs. 0.43 ± 0.24, P < 0.001) and greater cumulative tracer uptake, represented by the AUC at 175 and 630 seconds (P <0.001). No associations were found between dynamic parameters and preoperative prostate specific antigen level or Gleason score.

**Conclusions:**

In this pilot study, ^11^C-choline PET / CT demonstrated increased tracer uptake with higher values of static and dynamic parameters in areas of prostate cancer compared to areas of benign tissue. Larger studies are warranted to validate these results and examine the potential applicability of ^11^C-choline dynamic PET / CT for the diagnosis of prostate cancer.

## INTRODUCTION

Prostate cancer is the second most common cancer in men worldwide. An estimated 1.1 million new cases were recorded in 2012 (1). The diagnosis is based on direct systematic sampling of the prostate guided by transrectal ultrasound. This invasive method is necessary because gray-scale ultrasound itself is unreliable in detecting cancerous lesions (2). Nevertheless, the standard 12-core biopsy can miss up to one-third of cancers, resulting in repeated and more extensive biopsies (3).

Efforts have been made to develop new imaging modalities for the diagnosis and characterization of prostate cancer. This has become of paramount importance, given the emergence of new therapeutic approaches for localized prostate cancer (4).

Active surveillance and focal therapy are being applied more often in patients with low-risk localized prostate cancer, requiring improved tumor identification and classification. Better delineation of tumor extent would also assist surgical planning in patients with high-risk disease undergoing radical prostatectomy.

At present, patients with a suspicion of prostate cancer and negative biopsy findings are referred for multiparametric magnetic resonance imaging which has shown good sensitivity for high-grade tumors and an ability to detect anterior tumors, sometimes missed by systematic biopsy (5). However, its accuracy is often poor for low-volume or low-grade tumors (6), and despite the introduction of a scoring system (PIRADS), inter-reader variability remains a concern (7).

In addition, positron emission tomography (PET) has been examined as a potential means of visualizing primary prostate cancer and evaluating nodal extensions. By using a radiotracer for cancer diagnosis, clinicians can obtain information not only on the morphological appearance of the tumor but also on its biological behavior. Attention has recently been directed at ^11^C-choline PET / computed tomography (CT). Choline is a precursor of phosphatidylcholine, an essential element of cell membrane phospholipids. It has therefore been postulated that the ability of ^11^C- -choline PET to demonstrate cancerous lesions is related to the increased activity of choline kinase in tumor cells (8). Studies of the accuracy of ^11^C-choline PET / CT for the primary diagnosis of prostate cancer reported a sensitivity of 72% to 87% and a specificity of 62% to 84% (9–12). However, the vast majority evaluated radiotracer uptake semi-quantitatively, based on the standardized uptake value (SUV) at a single time point following injection. We speculated that tissue delineation could be improved by adding a dynamic (blood perfusion) phase to the PET / CT scan, which would allow for the assessment of multiple reactions involving choline uptake.

The aim of the present feasibility study was to examine the value of dynamic ^11^C-choline PET / CT for the primary diagnosis of prostate cancer in patients undergoing radical prostatectomy.

## MATERIALS AND METHODS

### Patients

A prospective case-series design was used. The study group consisted of consecutive patients with localized biopsy-proven adenocarcinoma of the prostate who underwent robot-assisted radical prostatectomy performed by a single surgeon (J.B.) at a tertiary medical center in 2012-2014. The local institutional review board approved the study. All patients provided written informed consent to participate in the study.

Data were collected on demographics, prostate-specific antigen (PSA) values, and preoperative biopsy results. Standard ^11^C-choline PET / CT was performed 2 months or less before surgery and at least 3 months after prostate biopsy.

### PET / CT protocol

Images were obtained using an integrated 8-slice PET-CT scanner (Discovery ST, GE Medical Systems, and Milwaukee, WI). The protocol included a dynamic PET acquisition limited to a single bed position and centered on the prostate, starting with the injection of 10-20mCi (370-740 MBq) of ^11^C-choline with a low-dose CT scan. After a scout view of the pelvis was obtained, the study centered on the prostate, with PET coverage of 15.3 cm. A non-diagnostic low-dose (30 mA) CT scan was acquired. ^11^C-choline was injected as a rapid bolus flushed with 50 cc saline 0.9% at a rate of 5.0 mL / sec using an automatic power injector (Dual-shot, Nemoto, Japan). The dynamic study was acquired as a 3D scan (matrix size 64 × 64, 3.27-slice thickness) consisting of 18 sequential frames of 5 seconds each followed by 9 frames of 60 seconds each. PET emission data after attenuation correction was reconstructed with a 3D OSEM algorithm (2 iterations, 20 subsets).

### Data processing and kinetic analysis

**Figure 1:**
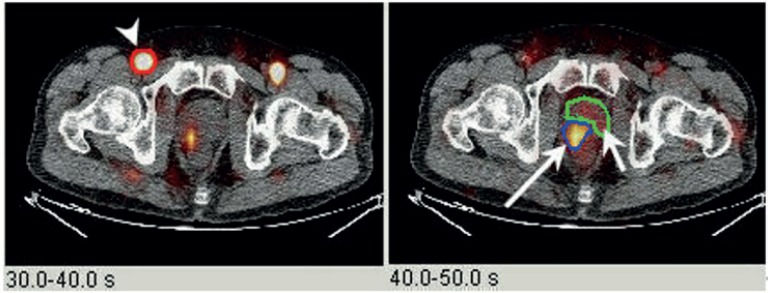
^11^C-choline PET / CT dynamic blood flow. Axial fused PET / CT sequential blood flow images (left: 30-40 seconds after injection; right: 40-50 seconds after injection) showing preferential arterial supply to the prostate tumor relative to background prostate tissue (arrowhead = iliac artery; large arrow = prostate cancer; small arrow = background prostate tissue).

**Figure 2:**
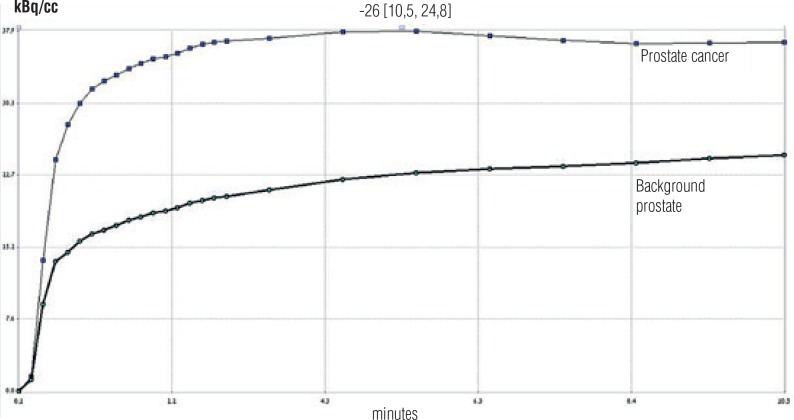
Linear fitted time-activity curves for prostate cancer (lighter line) and background prostate tissue (darker line).

Dynamic PET data analysis was performed using the PMOD software package (PMOD Technologies Ltd., Zurich, Switzerland). Imaging analysis was performed in a blinded fashion with respect to the histologic reference results. Oval volumes of interest (VOIs) were drawn manually over the suspected prostate tumor and the contralateral normal prostate tissue with a large artery (external iliac or femoral) within the frame (Figure-1). Time activity curves were generated for the mean activities of each VOI and fitted by linear regression function (Figure-2). A one-compartment model was used to assess tracer kinetics.

In pharmacokinetic modeling, tracer kinetics are separated into compartments and their interactions are represented by rate constants. The one-compartment pharmacokinetic model is used to simplify drug disposition in plasma and highly perfused tissue, under the assumption that there is an instantaneous mixing (drug behaves uniformly in a single unit) when the drug reaches the bloodstream. Changes in plasma concentration are equivalent to changes in tissue concentrations; dosing, sampling, and elimination occur from the central compartment. Total drug distribution is calculated as the ratio between the rate constant from the peripheral to the central compartment (K1) and the rate constant from the central to the peripheral compartment (K2).

The following parameters were used to evaluate suspected prostate tumor against background prostate tissue: transport constant (perfusion) K1 (mL / cm / min), rate constant K2 (l / min), total distribution volume (VT = K1 / K2, mL / ccm), area under the curve for tracer uptake during 175 seconds (AUC_175_) and 630 seconds (AUC_630_), SUV_avg_, and SUV_max_.

### Standard of reference

The surgical specimens served as the standard of reference. The harvested prostates were submitted for whole-mount histology. Axial sections of 3-5 mm were obtained in a plane perpendicular to a theoretical line between the base and the apex. Orientation was preserved by inking right and left margins with different colors (right-black, left-red) and by anatomical landmarks (i.e., seminal vesicle and urethra). Whole-mount sections of 4 μm each were stained with hematoxylin and eosin according to standard procedure. A Board-certified uropathologist (S.Z.) reviewed the slides and identified cancer foci by laterality and prostatic areas (base, mid-gland, and apex) and with respect to anatomic landmarks, as previously described.

### Matching PET / CT and histopathology

A single investigator (H.B), specialized in nuclear medicine, assessed the correspondence between the PET / CT images and histopathology findings. The most evident locations of the prostate tumor and normal prostate tissue segment were visually identified on the PET / CT images. The location of the prostate tumor was then confirmed from the pathological report with respect to anatomic landmarks. Thereafter, an oval region of interest (mean size 15 mm) was placed on the most evident tumor and in an area free of tumor.

## Statistical analysis

Continuous parameters are given as mean ± SD Static (SUV_max_, SUV_avg_) and dynamic (K1, K2, AUC and Vt) parameters of ^11^C choline PET / CT were compared between cancerous and non-cancerous regions using the Wilcoxon signed-rank test. A p value of < 0.05 was considered statistically significant.

## RESULTS

The study cohort included 24 patients of mean age 64 ± 5.4 years. The average interval from PET / CT scanning to surgery was 14 days (range, 3 to 61 days). Mean PSA level was 7.5 ± 5.3 ng / mL. On final surgical pathology, 16 patients (66%) had tumor confined to the prostate (AJCC stage II). Gleason score was 6 in 6 patients (25%), 7 in 16 patients (66%), and 9 in 2 patients (9%). A summary of the clinical and pathologic characteristics is presented in Table-1.

**Table 1:** Clinical and pathologic characteristics of 24 patients with prostate cancer

Characteristic		Value
Age (yr), mean (SD)		64.1 (5.4)
PSA (ng/ml), mean (SD)		7.5 (5.3)
Prostate volume (cm^3^), mean (SD)		52.7 (28.3)
**Final pathologic grade, n (%)**		
	Gleason (3+3)=6	6 (25)
	Gleason (3+4)=7	13 (55)
	Gleason (4+3)=7	3 (12)
	Gleason (4+5)=9	1 (4)
	Gleason (5+4)=9	1 (4)
**Final pathologic stage, n (%)**		
	pT2a	5 (21)
	pT2b	1 (4)
	pT2c	10 (42)
	pT3a	6 (25)
	pT3b	2 (8)

PSA-prostate-specific antigen

### Dynamic PET / CT parameters

Tracer uptake was observed in all 24 patients. SUV_avg_ and SUV_max_ were significantly higher in areas of cancerous tissue than areas of benign tissue (3.67 ± 2.7 vs. 2.08 ± 1.3 and 5.91 ± 4.4 vs. 3.71 ± 3.7, respectively, P < 0.001). There was a statistically significant difference in mean dynamic parameters of AUC_175_, AUC_630,_ and K1 between tumor and non-tumor zones (P < 0.001). Findings for constant of tracer washout from the tissue (K2) achieved borderline significance (P = 0.076). Table-2 shows the dynamic PET / CT parameters in benign and malignant tissue, and Figure-2 shows the linear fitted time-activity curves for prostate cancer and background prostate tissue. No associations were found between either Gleason scores or PSA levels and PET / CT parameters (P = 0.18 and P = 0.47, respectively).

**Table 2:** Comparison of ^11^C choline PET/CT parameters between prostate cancer and benign prostatic tissue.

Parameter (mean±SD)	Benign prostatic tissue	Prostate cancer	95% CI	*P* value
SUVavg	2.08±1.3	3.67±2.7	0.90-2.25	<0.001
SUVmax	3.66±3.7	5.91±4.4	1.65-2.83	<0.001
AUC_175_ (mL/ccm/min)	187.2±134	386.4±332	108.02-290.32	<0.001
AUC_630_ (mL/ccm/min)	964.8±644	1954.5±1734	488.44-1490-89	<0.001
Perfusion coefficient (K1) (l/ccm/min)	0.43±0.24	0.95±0.58	0.31-0.71	<0.001
Washout constant (K2) (1/min)	0.18±0.09	0.22±0.13	(-0.00-0.08)	0.076
Vt (K1/K2) (mL/ccm)	2.50±1.11	4.44±1.38	1.37-2.49	<0.001

**SUV_avg_**=standardized uptake value, average; **SUV_max_** = standardized uptake value, maximum; **AUC_175_** = area under the curve at 175 seconds; **AUC_630_** = area under the curve at 630 seconds, Vt-total distribution volume

## DISCUSSION

The present pilot study demonstrates the robust ability of dynamic ^11^C-choline PET / CT to differentiate cancerous from benign prostatic tissue. Statistically significant differences were found in both static and dynamic PET / CT parameters.

The moderate-range accuracy previously reported for diagnostic ^11^C-choline PET / CT in prostate cancer (9–12) was largely based on static parameters. However, while static parameters represent the sum of multiple reactions that occur during tracer uptake in a tissue, dynamic descriptors add sequential information that may assist in charac terizing the examined tissue. Two recent publications examined the diagnostic value of dynamic ^18^F-choline PET / CT for prostate cancer. In a study of 64 patients with prostate cancer, 19 with a primary diagnosis and 45 after biochemical recurrence, Takesh (13) reported a significant increase in mean dynamic constants (transport and rate) as well as in maximal SUV in cancerous areas compared to benign tissue. Similarly, Mathieu et al. (14) evaluated 39 patients with prostate cancer (18 with an initial diagnosis before any therapy) and found that tracer uptake was more rapid and intense in areas of cancerous lesions than in benign tissue. Although these studies provided encouraging results for the diagnostic ability of dynamic PET / CT, they were limited by a retrospective design, heterogenic patient sample (primary and post-treatment disease), and non-uniform standard reference.

The present study is distinctive in that it prospectively evaluated a cohort of consecutive patients, all with a diagnosis of localized prostate cancer who were primarily treated with radical prostatectomy. Whole-mount sections of the surgical specimen served as the reference standard for each patient. Furthermore, we used ^11^C choline as the radiotracer. ^11^C choline is harder to handle than ^18^F- -choline but it has a shorter half-life. We believe this is an important advantage because the rapid clearance in blood of ^11^C choline and its rapid uptake by prostate cells make it possible for clinicians to obtain early PET images without significant excretion of the tracer into the urine. The higher urinary excretion of ^18^F choline is a potential confounder as it may mask background activity (15).

We observed fast and intense uptake of ^11^C choline in cancerous tissue, with higher values of K1 in malignant regions in the prostate and a higher cumulative amount of tracer in the cancerous tissue, as demonstrated by the AUC. Contractor et al. (8) showed a relationship between increased ^11^C-choline uptake and overexpression of choline kinase. In their study, malignant lesions overexpressed choline kinase in both the cytoplasm and nucleus, and both SUV_avg_ and SUV_max_ were strongly associated with choline kinase staining.

Despite the rapid ^11^C-choline uptake and malignant nature of the tissue, we found no association of tracer uptake with the preoperative PSA level or Gleason score. This finding might be explained by the small size of the cohort and the small number of patients (n = 2) who had a high Gleason score on final pathologic analysis. Previous studies, using static ^11^C-choline PET / CT, showed limited ability to discriminate cancerous areas from areas of prostatitis (16). Dynamic ^11^C-choline PET / CT appears to better differentiate prostatitis from cancer, however the small number of patients with prostatitis in our study (n = 3) mandates further evaluation of this aspect.

Indeed, the small number of patients is the main limitation of our study. Another limitation is the fact that a single radiologist analyzed and interpreted the PET / CT images. Importantly, while novel imaging modalities using radiolabeled tracers with PSMA have shown promising results, the exact role of ^68^Ga-PSMA-PET / CT in primary prostate cancer is not yet entirely clear (17). Our preliminary results could potentially lead to a change in the way choline is being used as a tracer and improve the focal delineation of primary prostate cancer.

In conclusion, Dynamic ^11^C-choline PET / CT successfully distinguishes areas of prostate cancer from benign tissue based on values of static and dynamic parameters. Larger studies are warranted to validate our results and to examine the applicability of ^11^C-choline dynamic PET / CT in the primary diagnosis of prostate cancer.
